# Spatio-Temporal Dynamics of Asymptomatic Malaria: Bridging the Gap Between Annual Malaria Resurgences in a Sahelian Environment

**DOI:** 10.4269/ajtmh.17-0074

**Published:** 2017-10-30

**Authors:** Drissa Coulibaly, Mark A. Travassos, Youssouf Tolo, Matthew B. Laurens, Abdoulaye K. Kone, Karim Traore, Mody Sissoko, Amadou Niangaly, Issa Diarra, Modibo Daou, Boureima Guindo, Stanislas Rebaudet, Bourema Kouriba, Nadine Dessay, Renaud Piarroux, Christopher V. Plowe, Ogobara K. Doumbo, Mahamadou A. Thera, Jean Gaudart

**Affiliations:** 1 Department of Epidemiology of Parasitic Diseases, Malaria Research and Training Center, Faculty of Medicine and Dentistry, University of Sciences, Techniques and Technologies, Bamako, Mali;; 2 Division of Malaria Research, Institute for Global Health, University of Maryland School of Medicine, Baltimore, Maryland;; 3 Aix Marseille University, UMR MD3, Marseille, France;; 4 UMR ESPACE-DEV, IRD, Maison de la Télédétection, Montpellier, France;; 5 Aix Marseille University, IRD, INSERM, SESSTIM, Marseille, France

## Abstract

In areas of seasonal malaria transmission, the incidence rate of malaria infection is presumed to be near zero at the end of the dry season. Asymptomatic individuals may constitute a major parasite reservoir during this time. We conducted a longitudinal analysis of the spatio-temporal distribution of clinical malaria and asymptomatic parasitemia over time in a Malian town to highlight these malaria transmission dynamics. For a cohort of 300 rural children followed over 2009–2014, periodicity and phase shift between malaria and rainfall were determined by spectral analysis. Spatial risk clusters of clinical episodes or carriage were identified. A nested-case-control study was conducted to assess the parasite carriage factors. Malaria infection persisted over the entire year with seasonal peaks. High transmission periods began 2–3 months after the rains began. A cluster with a low risk of clinical malaria in the town center persisted in high and low transmission periods. Throughout 2009–2014, cluster locations did not vary from year to year. Asymptomatic and gametocyte carriage were persistent, even during low transmission periods. For high transmission periods, the ratio of asymptomatic to clinical cases was approximately 0.5, but was five times higher during low transmission periods. Clinical episodes at previous high transmission periods were a protective factor for asymptomatic carriage, but carrying parasites without symptoms at a previous high transmission period was a risk factor for asymptomatic carriage. Stable malaria transmission was associated with sustained asymptomatic carriage during dry seasons. Control strategies should target persistent low-level parasitemia clusters to interrupt transmission.

## INTRODUCTION

In the Sahel, where malaria transmission is seasonal, control programs aim to interrupt transmission or at least decrease malaria burden, demonstrating efficacy in several regions.^1–4^ These programs include deployment of rapid diagnosis tests (RDTs) and artemisinin-based combination therapy (ACT), the widespread distribution of insecticide-treated nets (ITNs), indoor residual spraying, intermittent preventive treatment (IPT) of pregnant women, and seasonal malaria chemoprophylaxis (SMC).

In Mali, malaria control strategies have included ACTs, widespread distribution of free long-lasting ITNs, diagnosis with free RDTs, and free medical care of children up to 5 years of age with uncomplicated or severe malaria. In the town of Bandiagara, Mali, recent studies document stable malaria incidence despite implementation of these control strategies in 2007.^5^

The high incidence of malaria in this rural area persists despite a 6-month dry season. Clinical cases of malaria occur only during the rainy season and the first half of the dry season, leaving a period of approximately3 months without clinical malaria episodes. In Sudan and Senegal, asymptomatic carriers of *Plasmodium* are present throughout the low transmission period.^6,7^ Long-term parasite carriage may be critical for parasite survival, and these infected individuals may constitute a major reservoir when environmental conditions are not favorable for mosquito development.^8^ Also, even in endemic areas, malaria transmission is not uniformly distributed; it can be patchy and dependent on factors, such as location of mosquito breeding sites and areas of clustered human habitations, which act as reservoirs of parasites.^9,10^ The persistence of malaria transmission in “hotspots,” such as a household or cluster of households may help the parasite to survive during the dry season.^10–12^ Strategies for detecting and targeting these hotspots, whether geographic or demographic, are important to reduce the local parasite reservoir and interrupting transmission.^13^

We hypothesized that annual resurgence of malaria transmission in Bandiagara is due to asymptomatic carriage of *P. falciparum* gametocytes and trophozoites and/or the existence of permanent transmission areas, which were undetected in previous studies. We assessed inter- and intra-annual spatio-temporal dynamics of malaria cases from 2009 to 2014 in Bandiagara, identifying spatial clusters and their temporal evolution between low and high transmission periods. We also identified parasite reservoirs, asymptomatic carriage evolution through low transmission periods, and risk factors for further clinical episodes of malaria.

## MATERIALS AND METHODS

This space-time dynamics of malaria distribution among children is part of a multiyear cohort study conducted by the malaria research and training center within the Bandiagara Malaria Project (BMP).

### Study setting.

Bandiagara is a semiurban area of approximately 19,000 inhabitants in 2014, situated in central Mali in West Africa and receives an annual rainfall of 600 mm. The rainy season extends from June to October and the dry season from November to May. Bandiagara’s small river Yamé stops flowing at the height of the dry and hot season (February–May) and transiently becomes a standing water body during the rainy season. *Anopheles gambiae* sensus stricto is the principal malaria vector. Malaria transmission is meso- to hyperendemic^14^ and highly seasonal.

Compared with less than one infecting bite/person/month from February to May, the transmission period peaks from August to September, with up to 60 infected mosquito bites/person/month. *Plasmodium falciparum* represents more than 95% of malaria infections.^15–17^ Children under 5 years of age receive free diagnostic testing and treatment of symptomatic malaria infection. Free bed nets have been distributed yearly since 2007. At each visit, participants were broadly questioned if they use protective measure including bednets. Pregnant women receive IPT free of charge according to the national malaria control policy.

### Study sample and design.

Three hundred children were recruited to be representative of the pediatric population aged 0–6 years of age, with 100 children in each of three age groups: 0–2, 3–4, and 5–6 years. After obtaining community permission, the study was publicized by a local radio broadcast. Parents were invited to accompany the children to the BMP research clinic to be screened for eligibility. Participants were enrolled on a first-come, first-served basis until the target number in each age strata was reached. Participants were followed from June 2009 to January 2015. During the study period, changes in participant households were not specifically investigated, but seemed to be rare. The loss to follow-up over 5 years was 23%, including four deaths.

### Inclusion and exclusion criteria.

Children were eligible for study inclusion if they met all of the following criteria: residency in Bandiagara town, general good health based on clinical evaluation, 0–6 years of age inclusive at the time of screening, written informed consent obtained from the parent/guardian, and availability to participate in follow-up for the duration of study. Exclusion criteria were as follows: simultaneous participation in an interventional clinical trial and chronic use of a medication with known antimalarial activity, such as trimethoprim-sulfamethoxazole.

### Study procedures.

Active surveillance consisted of scheduled monthly visits where participants were questioned for symptoms of malaria and examined. Finger prick blood was collected for malaria thick smear and hemoglobin level. Passive surveillance consisted of continuous availability of free, basic medical care at the BMP Research Clinic and Bandiagara District Hospital. If symptoms compatible with malaria were present, finger prick blood was collected for a diagnostic thick blood smear, which was read immediately. Uncomplicated malaria was treated with ACTs according to national guidelines.

Clinical malaria was defined based on clinical symptoms consistent with malaria (fever or history of fever, headaches, joint pain, vomiting, diarrhea, or abdominal pain) and the presence of malaria parasites at any density. Asymptomatic parasite carriage was defined as the presence of trophozoites or gametocytes by microscopy without any symptom after clinical evaluation. Gametocyte carriage was defined as the presence of gametocytes by microscopy, regardless of symptoms.

At enrollment, the household of each child was geo-referenced using a handheld global position system (Garmin^®^ Personal Navigator; accuracy approximately within 10 m).^5^ Rainfall data were obtained from the local weather station.

### Temporal and spatial cluster analysis and case mapping.

To assess time series (weekly incidence of symptomatic cases, weekly cumulated rainfall, monthly incidence of asymptomatic parasite carriage, and monthly incidence of gametocytes carriage), periodicity was first determined using multiscale spectral analyses (wavelet transform). The periodicity frequency test was based on a null hypothesis simulated by hidden Markov chains.^18,19^ Relationship and lag between malaria episodes and rainfall was assessed by estimating phase shift after wavelet transform. Asymptomatic parasite and gametocyte carriages were similarly assessed. We defined a low malaria transmission period (LTP) as the occurrence of fewer than two malaria clinical cases within two consecutive weeks and a high malaria transmission period (HTP) as two or more clinical cases within at least two consecutive weeks.

To assess the amount of rainfall associated with the total number of malaria symptomatic cases during the HTP, Spearman nonparametric correlation coefficients were estimated between different cumulative amounts of rainfall from the beginning of the rainy season and the total number of symptomatic malaria episodes.

Participant households were mapped according to their geographic coordinates. After aggregating data at the house block level, the number of malaria episodes per child was estimated for each block for each time-period (HTP and LTP) over the 5-year follow-up. The spatial distribution of clinical malaria incidence per time-period, as well as asymptomatic parasite and gametocyte carriages, were mapped by choropleth mapping at the house block level.

For each period, low and high-risk clusters were assessed using Kulldorff’s scanning approach (elliptic window, spatial Poisson model).^5,20^

### Parasite reservoir assessment.

To identify potential parasite reservoirs for low and high transmission periods, we assessed risk factors associated with clinical malaria and asymptomatic and gametocyte carriage. Controls were individuals within the same time period with negative malaria smears.

After a nested case–control design,^21^ each case was matched by year, transmission period, bed-net use, and neighborhood to four controls. Multivariate conditional logistic regression analyses were performed to evaluate potential risk factors associated with clinical malaria and asymptomatic and gametocyte carriage and to estimate the corresponding adjusted odds ratios (ORa). The effect of past parasite carriage was assessed by estimating ORa associated with past clinical episodes and with past asymptomatic and past gametocyte carriages at previous low or high transmission periods (LTPs or HTPs).

Analyses were adjusted for sex, level of hemoglobin at visit, hemoglobin type, G6PD deficiency, and age at visit. Variable selection was provided by a stepwise approach.

### Laboratory assays.

Malaria thick smears were Giemsa-stained, and parasites were counted against 300 leukocytes to give parasite counts/mm^3^, assuming a leukocyte count of 7,500/mm^3^. Standard operating procedures were developed to ensure uniform and high-quality malaria smears, including training and qualifying malaria microscopists. Haemoglobin type was determined by high-performance liquid chromatography (D-10 instrument; Bio-Rad). Restriction fragment length polymorphism analysis was used to identify the (A^−^) allele of the G6PD deficiency, as previously described.^15^ Haemoglobin determinations were made using Hemocue haemoglobin analyzers (Hemocue Inc., Cypress, CA).

### Ethical compliance.

The study was conducted in compliance with the International Conference on Harmonization Good Clinical Practices, the Declaration of Helsinki, and regulatory requirements of Mali. Details on the consent and the protocol approval process have been described elsewhere.^17^

### Statistical consideration.

Multiscale spectral analyses were performed using MATLAB R2013a software (^©^The MathWorks Inc, Natick, MA) with the Wavelet_EETS tool (http://www.biologie.ens.fr/∼cazelles/bernard/Research.html). Maps have been provided by Philcarto 6.72 software (^©^P Waniez, CNRS and Université de Bordeaux, France).^22^ Hotspots were analyzed using the SaTScan 8.2.1 software (^©^M Kulldorff, Harvard Medical School, Boston and Information Management Services Inc, Calverton, MA). All other statistics were performed using R version 3.1.3 (^©^The R Foundation for Statistical Computing, Vienna, Austria). Figures were formatted using Gimp 2.8.14 software (^©^S Kimball, P Mattis and The Gimp Team).

## RESULTS

### Malaria case incidence.

**Figure 1.**
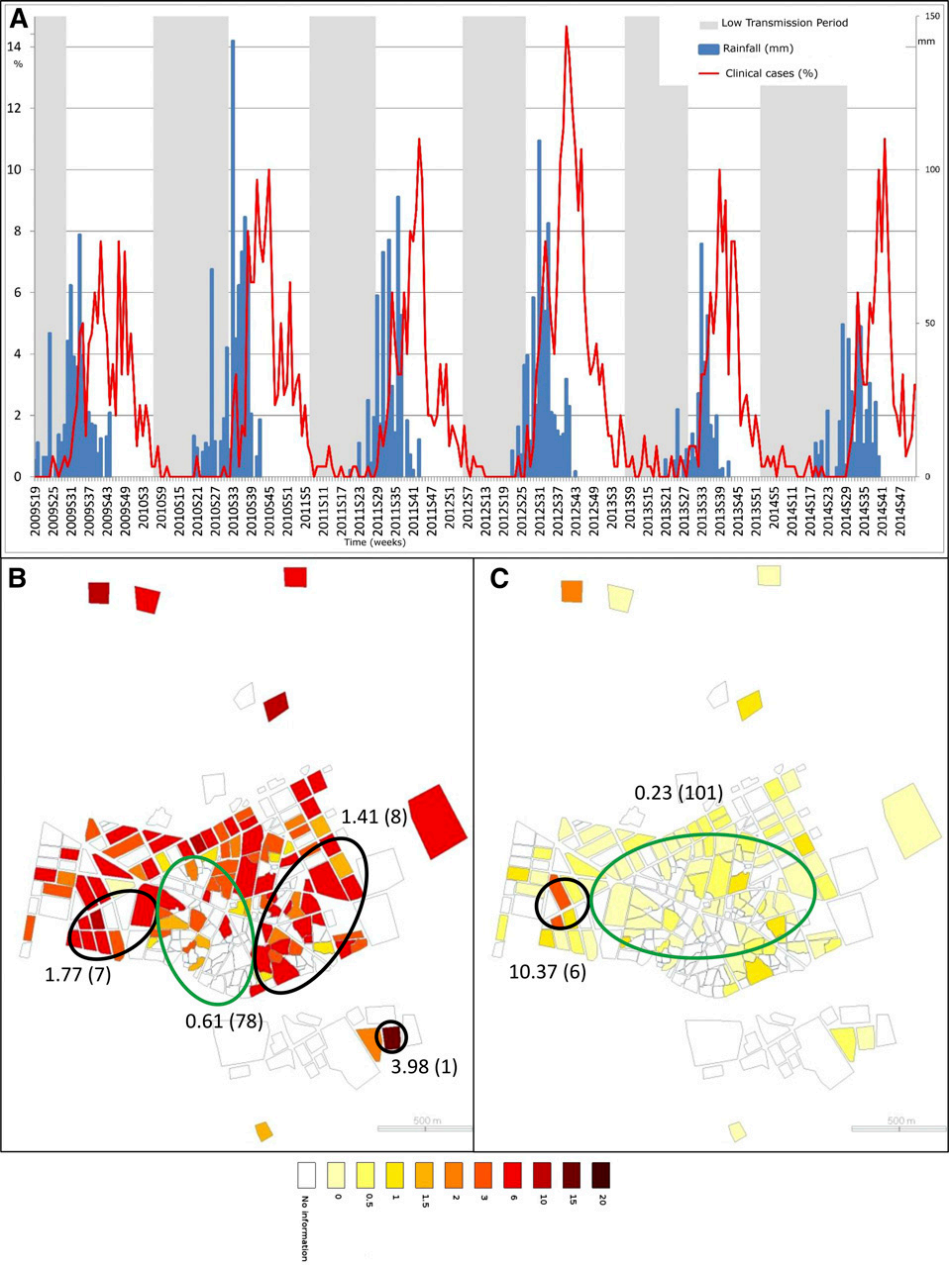
Spatial and temporal pattern of malaria cases. (**A**) represents the weekly time series of clinical case incidence per 100 children (red). Weekly rainfall is presented in blue. Low and high transmission period are presented, respectively, in grey and white. Choropleth maps of clinical case incidences, (**B** and **C**), are presented at the neighborhood level, respectively, for high and low transmission periods (through 2009–2014). Green ellipses represent low risk cluster (according to Kulldorff’s definition) and black ones, high risk clusters. Estimated relative risks of each cluster are presented (as well as the number of neighborhood within each cluster, between brackets). This figure appears in color at www.ajtmh.org.

**Figure 2.**
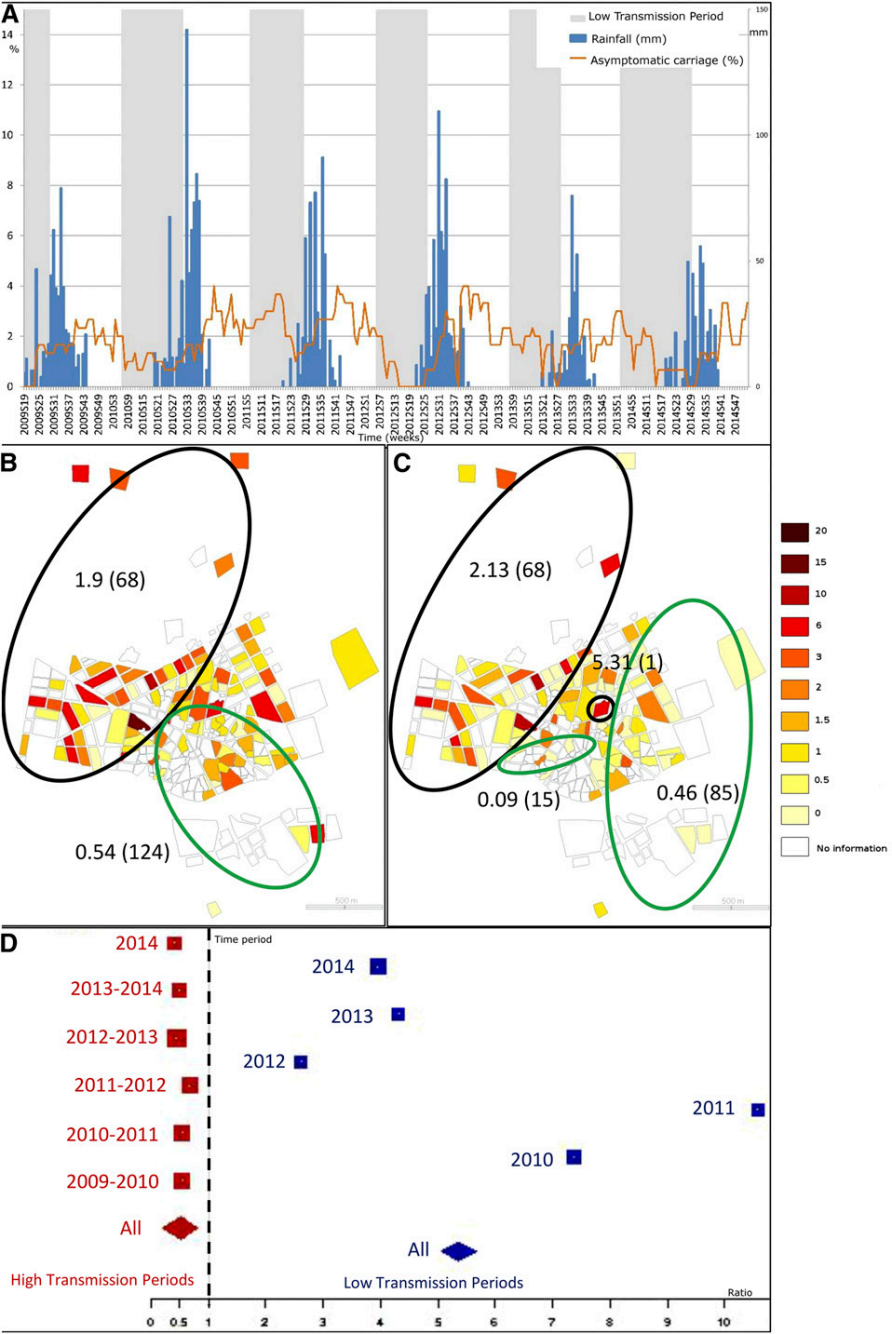
Spatial and temporal pattern of asymptomatic carriage. (**A**) represents the weekly time series of asymptomatic carriage incidence per 100 children (orange). Weekly rainfall is presented in blue. Low and high transmission periods are presented, respectively, in grey and white. Choropleth maps of asymptomatic incidences, (**B** and **C**), are presented at the neighborhood level, respectively, for high and low transmission periods (through 2009–2014). Green ellipses represent low risk clusters (according to Kulldorff’s definition) and black ones, high risk clusters. Estimated relative risks of each cluster are presented (as well as the number of neighborhoods within each cluster, between brackets). (**D**) represents the asymptomatic carriage ratios by clinical cases for each high and low transmission period (red and blue, respectively). Each square is proportional to each period length. The diamonds represent the ratios for the high and low transmission periods (red and blue, respectively). This figure appears in color at www.ajtmh.org.

Time-series analysis showed a persistence of malaria across all years of the study period (Figure 1A), as well as similar malaria incidence across all years without any decreasing trend. High transmission periods (HTPs) began weeks after rainy season onset and ceased 2–3 months after the rains stopped. Assessment of periodicity and lag between malaria case incidence and rainfall by spectral analysis confirmed a significant annual periodicity of both malaria incidence and rainfall (Figure 2A and B), with very few clinical cases diagnosed during the second half of each dry season and the first weeks of each rainy season. The phase analysis (Figure 2C) showed a phase shift of 3 months between rainfall and clinical cases (π/2). Cumulative rain within the first 5 weeks was the rain amount most closely correlated with the total amount of clinical cases during the following high transmission season (*r* = 0.89, *P* = 0.03). This also explains the highest peak of malaria incidence, in 2012, where the rainfall was lower than previous years, but most of the rain occurred within the first week.

Within high transmission periods, spatial cluster analysis revealed a cluster at low risk for clinical malaria cases (Figure 1B) with a relative risk (RR) = 0.61 (*P* = 0.001), located in the town center and including 78 households. Three significant high-risk clusters were also identified, located in the west (RR = 1.77, *P* = 0.001, seven locations), the east (RR = 1.41, *P* = 0.001, eight locations), and the southeast (RR = 3.98, *P* = 0.002, one location). Within low transmission periods (Figure 1C), a large low-risk cluster was identified in the town center (RR = 0.23, *P* = 0.001, 101 locations), as well as a high-risk cluster in the west (RR = 10.37, *P* = 0.001, six locations). Furthermore, throughout the study, locations of clusters did not greatly change from 1 year to another.

### Malaria reservoir assessment.

**Figure 3.**
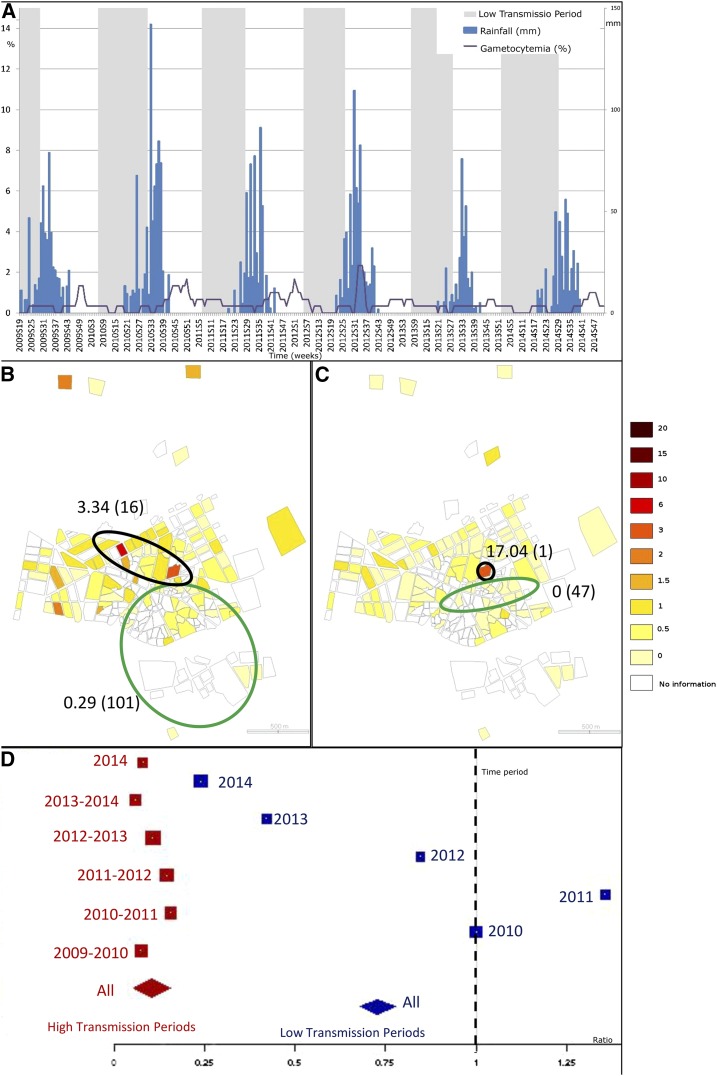
Spatial and temporal pattern of gametocytemia. (**A**) represents the weekly time series of gametocytemia incidence per 100 children (purple). Weekly rainfall is presented in blue. Low and high transmission period are presented, respectively, in gray and white. Choropleth maps of gametocytemia incidences, (**B** and **C**), are presented at the neighborhood level, respectively, for high and low transmission periods (through 2009–2014). Green ellipses represent low risk clusters (according to Kulldorff’s definition) and black ones, high risk clusters. Estimated relative risks of each cluster are presented (as well as the number of neighborhoods within each cluster, between brackets). (**D**) represents the positive gametocytemia ratios by clinical cases for each high and low transmission period (red and blue, respectively). Each square is proportional to each period length. The diamonds represent the ratios for the high and low transmission periods (red and blue, respectively). This figure appears in color at www.ajtmh.org.

**Figure 4.**
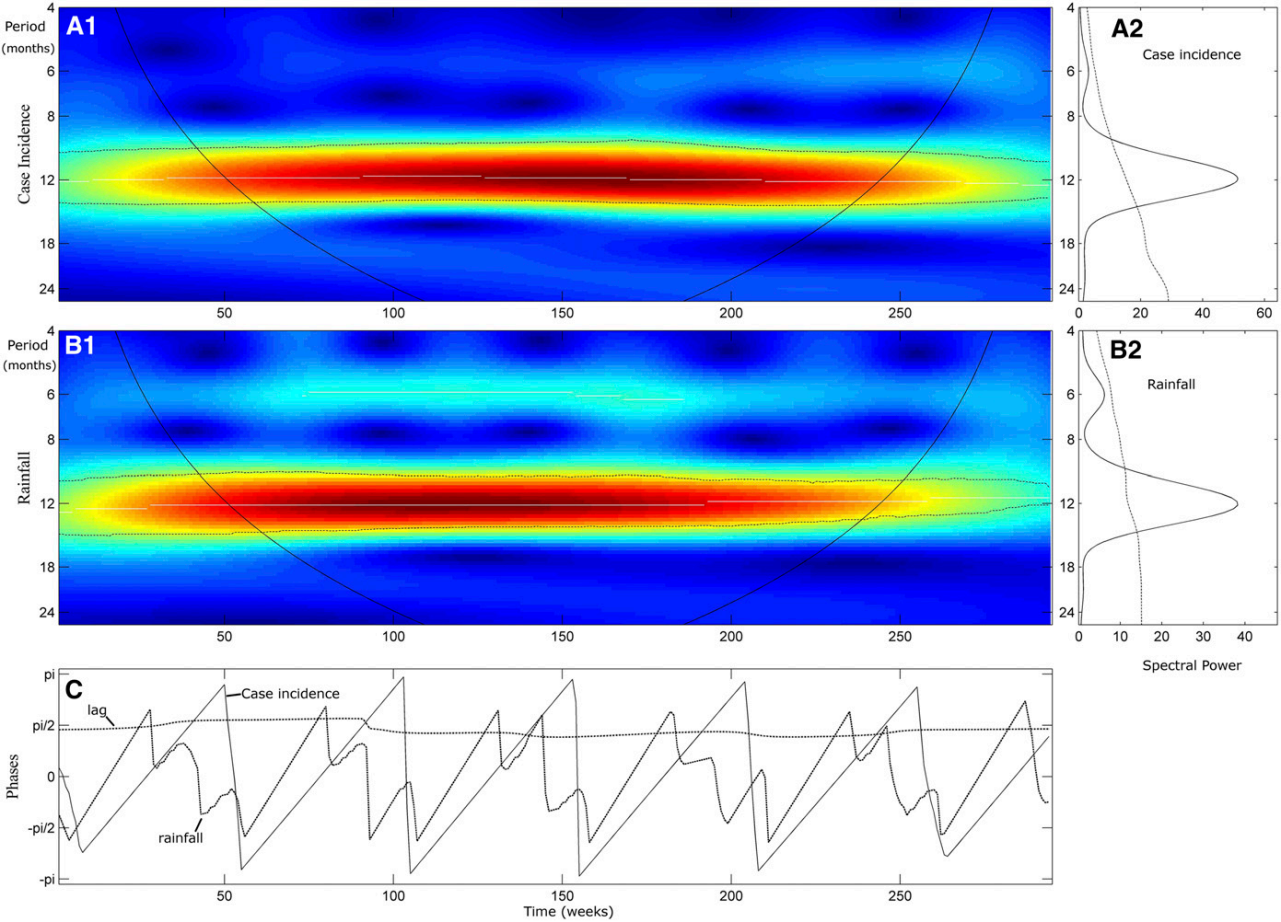
Wavelet analysis of weekly clinical malaria incidence and weekly rainfall. The different panels comprise each time series analysis. The left panels (**A1** and **B1**) are the wavelet power spectrum of the square root transformed and normalized series (2009–2014). The color code for power values ranges from dark blue (low values) to dark red (high values). The dotted black lines show the statistically significant area (threshold of 95% CI). The white lines represent the maxima of the wavelet power spectrum, and the black curves indicate the cone of influence that delimits the region not influenced by edge effects. The right panels (**A2** and **B2**) correspond to the mean spectrum (black solid line) with its threshold value of 95% CI (dotted black line) for the aggregate time series. The third raw panel (**C**) represents the phase evolution of the two time series, using wavelet analysis. The black dotted line represents the phase differences (lag) between the two series. This figure appears in color at www.ajtmh.org.

Time series showed persistence of both asymptomatic (Figure 3A) and gametocyte (Figure 4A) carriage, even during low transmission periods. Both asymptomatic and gametocyte carriage resurged and plummeted with a clear annual periodicity (Supplemental Figures 1 and 2), but without time lags before each clinical case time series. Furthermore, for each high transmission period, the ratio of asymptomatic carriers to clinical cases (Figure 3D) was approximately 0.5 (median ratio = 0.511, range [0.406, 0.677]). By contrast, this ratio was inverted during all low transmission periods, where the number of asymptomatic carriers was approximately five times the number of clinical cases (median ratio = 4.491, range [2.615, 10.571]). Regardless of time period, gametocyte carriers were outnumbered by clinical cases (high transmission period: median ratio = 0.092, range [0.057, 0.155]; low transmission period: median ratio = 0.923, range [0.238, 1.357]), but the ratio was higher within low versus high transmission periods (Figure 4D).

Throughout high transmission periods (Figures 3B and 4), both asymptomatic and gametocyte carriage had one low-risk cluster each (respectively RR = 0.54, 124 locations, *P* = 0.001; RR = 0.29, 101 locations, *P* = 0.001) located around the same area at the center and the south, and one high-risk cluster each (respectively RR = 1.9, 68 locations, *P* = 0.001; RR = 3.34, 16 locations, *P* = 0.001), located at the west and north for asymptomatic carriers, and at the center and north for gametocyte carriers. Asymptomatic and gametocyte risk clusters differed for the low transmission periods. Two low-risk clusters for asymptomatic carriers (Figure 3C) were identified at the south-central and at the east ( RR = 0.09, 15 locations, *P* = 0.007; RR = 0.46, 85 locations, *P* = 0.001, respectively). Two high-risk clusters for asymptomatic carriers included a large one (RR = 2.13, *P* = 0.001) accounting for 68 locations and located similar to the HTP high-risk cluster (west and north) and a small one with a single location at the town center (RR = 5.31, *P* = 0.001). Gametocyte carrier risk included, throughout low transmission periods, two small clusters: a low-risk one (RR = 0, *P* = 0.001) located at the town center near a high-risk cluster (RR = 17.04, *P* = 0.001) centered on a single location (Figure 4C).

### Risk factors associated with malaria.

Multivariate nested case-control analyses (Table 1), matched by time-period, bed net, and neighborhood, confirmed that age was a protective factor for clinical episodes during high and low transmission periods (respectively ORa = 0.02.10^−3^ CI 95% [0.01.10^−3^, 0.1.10^−3^]; ORa = 0.002 [0.03.10^−3^, 0.09]). The number of clinical episodes at the previous high transmission period was also a protective factor for clinical episodes during the following high transmission one (ORa = 0.61 [0.38, 0.96]).

**Table 1:** Risk factors associated with clinical malaria episodes

Risk factors associated with clinical malaria episodes in the high transmission period (HTP)						
Variables	Case (*N* = 1,507)	Control (*N* = 7,535)	OR1 CI 95% [ ]	*P*	ORa2 CI 95% [ ]	*P*
Age visit mean (SD)	5.9 (2.17)	7.75 (1.93)	0.03.10^−3^ [0.01.10^−3^; 0.1.10^−3^]	< 0.001	0.02.10^−3^ [0.01.10^−3^; 0.1.10^−3^]^3^	< 0.001
CE_HTP mean (SD)	1.45 (1.36)	1.38 (1.3)	1.06 [1.01; 1.11]	0.025	0.61 [0.38; 0.96]^3^	0.03
Gam_LTP mean (SD)	0.04 (0.23)	0.09 (0.32)	0.5 [0.39; 0.65]	< 0.001	–	–
Gam_HTP mean (SD)	0.12 (0.42)	0.19 (0.54)	0.7 [0.61; 0.81]	< 0.001	–	–
ASPara_LTP mean (SD)	0.24 (0.76)	0.56 (1.21)	0.68 [0.63; 0.73]	< 0.001	–	–
ASPara_HTP mean (SD)	0.54 (0.98)	1.03 (1.49)	0.69 [0.65; 0.73]	< 0.001	–	–
Risk factors associated with clinical malaria episodes in the low transmission period (LTP)						
Variables	Case (*N* = 39)	Control (*N* = 195)	OR1 CI 95% [ ]	*P*	ORa2 CI 95% [ ]	*P*
Age visit mean (SD)	5.94 (2.06)	7.84 (1.93)	0.003 [0.1.10^−3^; 0.11]	0.001	0.002 [0.03.10^−3^; 0.09]^3^	0.002
CE_LTP mean (SD)	0.26 (0.55)	0.11 (0.35)	2.83 [1.15; 7.0]	0.024	–	–
CE_HTP mean (SD)	2.05 (1.41)	1.21 (1.26)	1.92 [1.35; 2.74]	< 0.001	–	–
Gam_LTP mean (SD)	0	0.16 (0.44)	–	–	–	–
ASPara_LTP mean (SD)	0.39 (0.82)	0.94 (1.44)	0.59 [0.39; 0.88]	0.009	–	–
ASPara_HTP mean (SD)	1.49 (1.86)	2.66 (1.98)	0.62 [0.48; 0.8]	< 0.001	2.12 [0.082; 54.83]^3^	0.65

Age visit = age at visit; ASPara_HTP = number of asymptomatic parasite carriage cases on previous HTP; ASPara_LTP = number of asymptomatic parasite carriage cases on previous Asy.LTP; CE_HTP = number of clinical malaria episodes in previous Clin.HTP; CE_LTP = number of clinical malaria episodes on previous LTP; Gam_HTP = number of positive gametocyte cases on previous HTP; Gam_LTP = number of positive gametocyte cases on previous LTP Gam.HTP; OR1 = odds ratio from conditional logistic regression (matching to the three designated variables); ORa2 = adjusted odds ratio from conditional logistic regression (matching to the three designated variables) and result of multivariate step by step analysis.

The risk of parasite carriage (either asymptomatically or as gametocytes) was also significantly reduced by age throughout high and low transmission periods (see Table 2). Adjusted by age, and according to time-period, bed-net and neighborhood, the number of clinical episodes at previous high transmission periods was a protective factor for asymptomatic carriage during the subsequent low transmission period (ORa = 0.26 [0.07, 0.93]), but carrying parasites without symptoms at a previous high transmission period was a risk factor for asymptomatic carriage during a high transmission period (ORa = 2.67 [1.52, 4.69]; Table 2).

**Table 2:** Risk factors associated with asymptomatic parasite or gametocyte carriage

Risk factors associated with asymptomatic parasite carriage in the HTP						
Variables	Case (*N* = 680)	Control (*N* = 3,400)	OR1 CI 95% [ ]	*P*	ORa2 CI 95% [ ]	*P*
PCRg6pd	133	498	1.45 [1.16; 1.80]	< 0.001	–	–
Age visit mean (SD)	6.2 (2.15)	7.76 (1.88)	0.001 [0.001; 0.003]	< 0.001	0.002 [0.001; 0.003]^2^	< 0.001
Gam_HTP mean (SD)	0.27 (0.67)	0.2 (0.57)	1.22 [1.07; 1.39]	0.003	–	–
ASPara _HTP mean (SD)	1.25 (1.63)	1.09 (1.58)	1.08 [1.02; 1.14]	0.007	2.67 [1.52; 4.69]^2^	< 0.001
Risk factors associated with asymptomatic parasite carriage in the LTP						
Variables	Case (*N* = 132)	Control (*N* = 660)	OR1 CI 95% [ ]	*P*	ORa2 CI 95% [ ]	*P*
Age visit mean (SD)	5.39 (2.39)	7.55 (2.14)	0.1.10^−3^ [0.1.10^−5^; 0.002]	< 0.001	0.03.10^−3^ [0.1.10^−5^; 0.001]^3^	< 0.001
CE_HTP mean (SD)	1.25 (1.29)	1.03 (1.04)	1.23 [1.03; 1.46]	0.021	0.26 [0.07; 0.93]^3^	0.038
ASPara_LTP mean (SD)	0.5 (1.15)	1.02 (1.57)	0.65 [0.54; 0.79]	< 0.001	–	–
ASPara_HTP mean (SD)	1.29 (1.51)	2.61 (2.01)	0.56 [0.48; 0.866]	< 0.001	–	–
Risk factors associated with gametocyte carriage in the HTP						
Variables	Case (*N* = 173)	Control (*N* = 865)	OR1 CI 95% [ ]	*P*	ORa2 CI 95% [ ]	*P*
Clinical signs	54	522	0.28 [0.19; 0.41]	< 0.001	0.16 [0.09; 0.31]	
Age visit mean (SD)	6.06 (2.08)	7.56 (1.77)	0.005 [0.002; 0.02]	< 0.001	0.002 [0.001; 0.01]^3^	< 0.001
Risk factors associated with gametocyte carriage in the LTP						
Variables	Case (*N* = 53)	Control (*N* = 159)	OR1 CI 95% [ ]	*P*	ORa2 CI 95% [ ]	*P*
Age visit mean (SD)	5.86 (2.24)	7.54 (1.93)	0.002 [0.1.10^−3^; 0.05]	< 0.001	0.002 [0.1.10^−3^; 0.04]^3^	< 0.001
CE_HTP mean (SD)	1.21 (1.06)	0.91 (1.02)	1.55 [1.05; 2.29]	0.028	0.35 [0.03; 3.54]^3^	0.372

Age visit = age at visit; ASPara_HTP = number of asymptomatic parasite carriage cases on previous HTP; ASPara_LTP = number of asymptomatic parasite carriage cases on previous LTP à changer en Asy.LTP; CE_HTP = number of clinical malaria episodes in previous HTP; CE_LTP = number of clinical malaria episodes on previous LTP; Gam_HTP = number of positive gametocyte cases on previous HTP; Gam_LTP = number of positive gametocyte cases on previous LTP Gam.HTP; OR1 = odds ratio from conditional logistic regression (matching to the three designated variables); ORa2 = adjusted odds ratio from conditional logistic regression (matching to the three designated variables) and result of multivariate step by step analysis.

## DISCUSSION

By analyzing the spatio-temporal dynamics of clinical and asymptomatic malaria in Bandiagara, this study explored reasons for *P. falciparum* persistence through the dry season. The 3-month phase shift between rain and clinical case onset along with the slow decrease in transmission until the middle of the dry season indicates that rainy season and high transmission season are not completely coincident, nor are the dry season and low transmission season. Despite no clinical episodes or gametocyte carriage during some weeks, asymptomatic carriage persisted in the community, with a ratio to clinical episodes from high to low transmission periods of about 0.5 to 5, respectively. Throughout the entire dry season, malaria transmission was low, and asymptomatic carriers were the only reservoir of parasites during this time. Mass drug administration during low transmission periods to reduce parasitic reservoirs could reduce the number of cases during the following high transmission season.

The absence of a lag between the clinical case and asymptomatic time series suggests that asymptomatic carriage may provide the reservoir leading to rainy season malaria cases and each annual malaria epidemic. During the high transmission period, the number of asymptomatic carriers is relatively low, and clinical cases may be the main parasite reservoir. These results support adoption of SMC strategies,^23^ specifically starting 2–3 months after the rain begins, not concomitantly with it.

For each transmission period, clinical case clusters and parasite carriage clusters (high or low risk) differed, but remained stable across seasons. Clusters at high risk for symptomatic malaria were distributed in the western and the eastern parts of town not far from the Yamé River. Residence in the town center was protective against symptomatic malaria, probably due to the previously described barrier effect of households.^24^ The marked spatial heterogeneity of malaria incidence across the study period may be due to the location of mosquito breeding sites.^5^ Parasite carriage clusters (asymptomatic or gametocytes) were mostly located in the central and western parts of town and remained stable across the 5 years of the study. Locations where parasites persisted during low transmission periods should be targeted with strategies focused on eliminating parasite reservoirs.

In an adjusted analysis, experiencing clinical episodes was protective against future clinical episodes and asymptomatic carriage. This may be explained by several hypotheses: first, appropriate treatment of clinical episodes with ACTs would reduce parasite carriage in the following months. ACT combinations are not only effective against parasite asexual stages, but can also have an impact on the sexual stages transmitted to mosquitoes,^25^ reducing malaria transmission. Second, a clinical episode in a child could lead to an improved parental adherence to prevention strategies, such as bed-net use (“social immunization”). Third, experiencing a clinical episode could also act as individual “premunition”, reducing the risk of malaria for the next transmission period.

Age was a significant protective factor against parasite carriage, as has been shown previously,^26^ likely because of the acquisition of immunity with age (premunition). Previous work has shown that parasite carriage is protective against new clinical episodes.^27^ The immunity level of an individual against infection depends on age and history of exposure.^8^ Asymptomatic carriage thus has a potential benefit for both the host and the parasite: the host can maintain immunity while the parasite persists through adverse environmental condition periods.

Diagnosing asymptomatic malaria is not straightforward because of the absence of clinical signs. Microscopic detection has low sensitivity compared with Polymerase chain reaction, and we may have underestimated the prevalence of asymptomatic carriage.^25,28^ Scaling up efforts to improve the characterization of asymptomatic malaria in endemic regions and establishing a standard case definition should be a priority.^29^

Parasite carriage allows for undetected circulation during interepidemic periods, when environmental local conditions are less favorable to mosquitoes. In this context, the beginning of the rainy season initiates or boosts malaria transmission in conjunction with mosquito breeding site proliferation. This may explain malaria persistence through interepidemic periods and the periodical revival of symptomatic disease when environmental conditions favor mosquito development.

At each visit, participants were routinely asked if they used protective measures, which included bednets. They reported the use of long-lasting, insecticide-treated bed nets (LLINs).

LLINs are provided for free to children and pregnant woman by Mali’s National Malaria Control Program. This did not change during the study period. Furthermore, the use of LLINs was taken into account (as a matching factor) in the nested case control study.

A limitation of this study was the lack of precise assessment of the parasite reservoir, both within the entire population, including adult carriers, and within the vector population, lacking entomological assessments. Indeed, estimations of the parasitological “biomass”, location, and circulation need to be addressed for effective new elimination strategies.

The current suite of interventions has proven insufficient to eliminate malaria from many areas in sub-Saharan Africa. For malaria elimination, we need additional strategies.^8^ Rapid treatment, the screening and treatment of asymptomatic carriage, the treatment of gametocyte carriage, the detection and management of habitats favoring the persistence of *Anopheles* mosquitoes, and mass or focused screening and treatment should be considered. The precise dynamic of parasite persistence through low transmission periods needs additional scrutiny, as well as the dynamic of malaria resurgence after rainfall. The analytic approach that we have used here has the potential to provide considerable insight into these dynamics, as well as effective malaria control strategies.

## Supplementary Material

Supplemental Figure.
