# Supplementary material for: Spatio-Temporal Dynamics of Asymptomatic Malaria: Bridging the Gap Between Annual Malaria Resurgences in a Sahelian Environment
Source: Am J Trop Med Hyg. 2017 Oct 30;97(6):1761–9. doi: 10.4269/ajtmh.17-0074 (PMC5805033; doi:10.4269/ajtmh.17-0074)
Supplement: Supplementary file 1 [file tpmd170074.SD1.pdf]

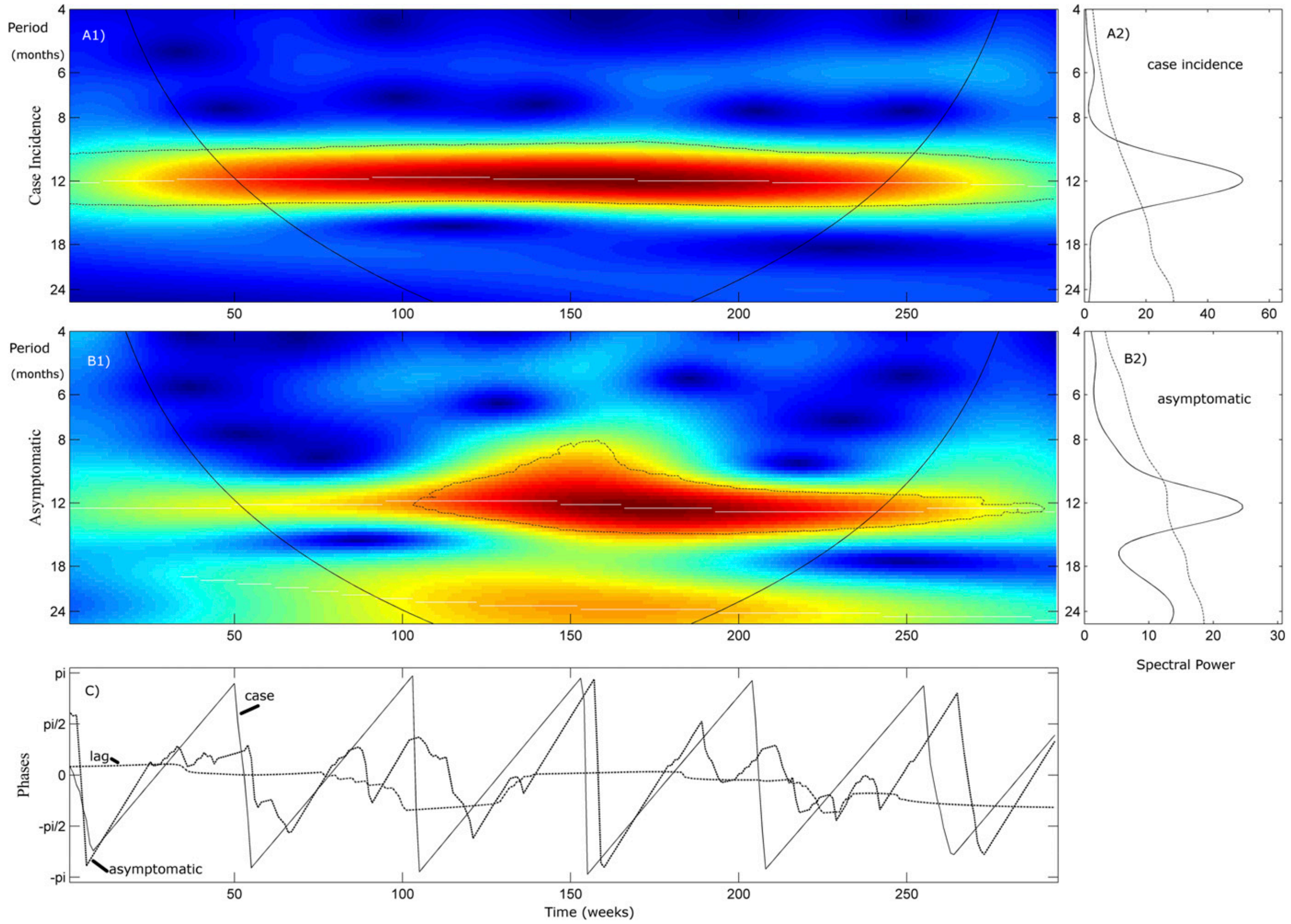

Supplemental Figure 1. Wavelet analysis of weekly clinical malaria incidence and asymptomatic carriage. The different panels comprise each time series analysis. The left panels (**A1** and **B1**) are the wavelet power spectrum of the square root transformed and normalized series (2009–2014). The color code for power values ranges from dark blue (low values) to dark red (high values). The dotted black lines show the statistically significant area (threshold of 95% CI). The white lines represent the maxima of the wavelet power spectrum, and the black curves indicate the cone of influence that delimits the region not influenced by edge effects. The right panels (**A2** and **B2**) correspond to the mean spectrum (black solid line) with its threshold value of 95% CI (dotted black line) for the aggregate time series. The third raw panel (**C**) represents the phase evolution of the two time series, using wavelet analysis. The black dotted line represents the phase differences (lag) between the two series.

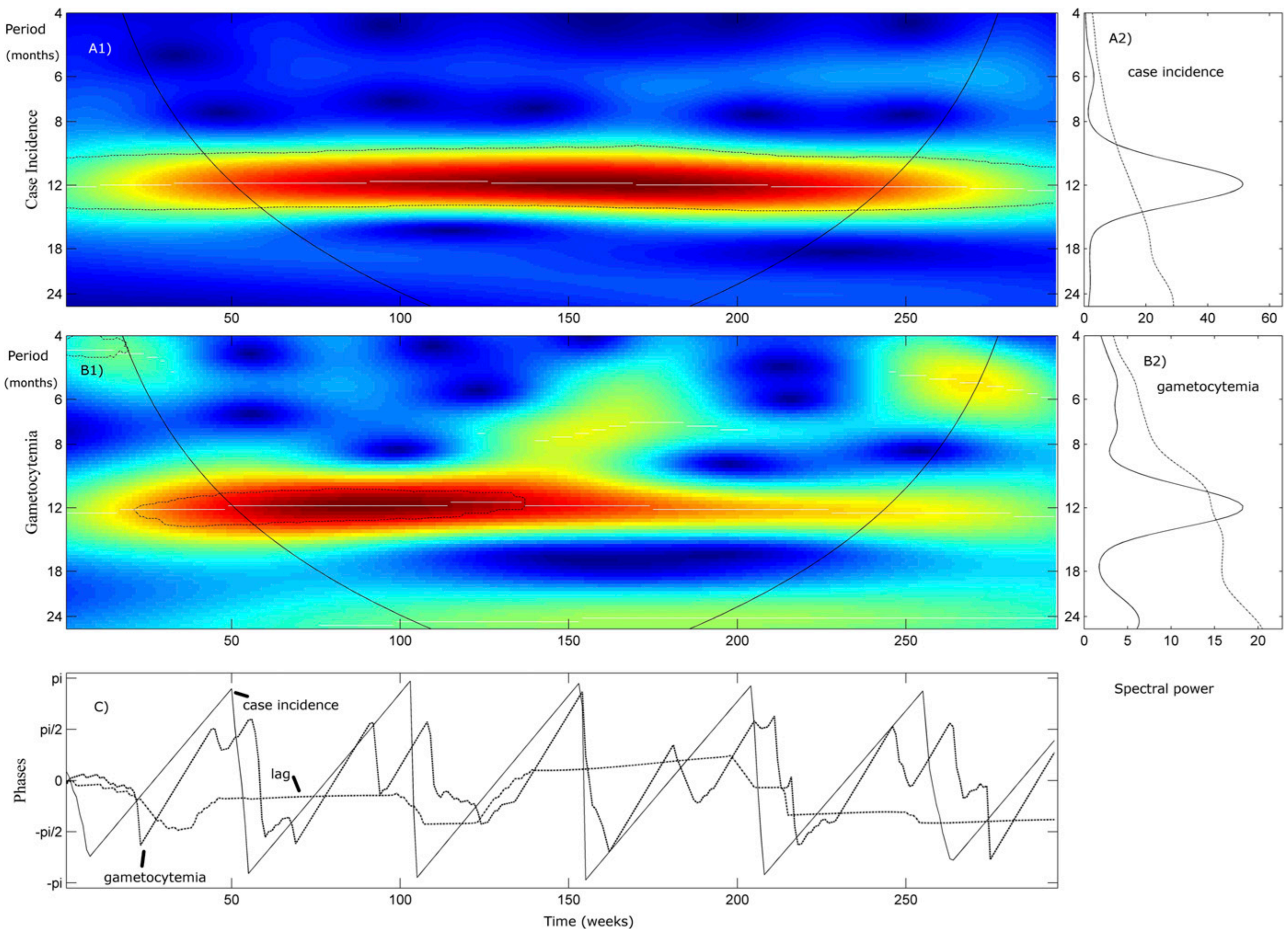

SUPPLEMENTAL FIGURE 2. Wavelet analysis of weekly clinical malaria incidence and gametocytemia. The different panels comprise each time series analysis. The left panels (**A1** and **B1**) are the wavelet power spectrum of the square root transformed and normalized series (2009–2014). The color code for power values ranges from dark blue (low values) to dark red (high values). The dotted black lines show the statistically significant area (threshold of 95% CI). The white lines represent the maxima of the wavelet power spectrum, and the black curves indicate the cone of influence that delimits the region not influenced by edge effects. The right panels (**A2** and **B2**) correspond to the mean spectrum (black solid line) with its threshold value of 95% CI (dotted black line) for the aggregate time series. The third raw panel (**C**) represents the phase evolution of the two time series, using wavelet analysis. The black dotted line represents the phase differences (lag) between the two series.
